# Challenges in the Pharmacotherapeutic Management of Pediatric Asthma

**DOI:** 10.3390/ph15121581

**Published:** 2022-12-18

**Authors:** Ileana Ioniuc, Ingrith Miron, Vasile Valeriu Lupu, Iuliana Magdalena Starcea, Alice Azoicai, Monica Alexoae, Anca Adam Raileanu, Felicia Dragan, Ancuta Lupu

**Affiliations:** 1 Pediatrics, “Grigore T. Popa” University of Medicine and Pharmacy, 700115 Iasi, Romania; 2 Faculty of Medicine and Pharmacy, University of Oradea, 410087 Oradea, Romania

**Keywords:** pediatric bronchial asthma, GINA, child, adolescent, treatment

## Abstract

Bronchial asthma is one of the most common chronic conditions in pediatric practice, with increasing prevalence hampered by poor socioeconomic impacts, leading to major public health issues. Considered as a complex heterogeneous syndrome, not a single disease, the management of the disease is a real challenge, impacting medical staff, patients and caregivers. Over the decades, a significant number of diagnostic and treatment regimen have been developed to achieve good standards, sustaining balanced control of the disease. This paper attempts a review on the establishment of new trends in the management of bronchial asthma in the pediatric age group.

## 1. Introduction

Bronchial asthma is one of the most common chronic pathologies in pediatric practice. It affects approximately 5.5 million children in the European Union with an average prevalence of 10% across member countries. Despite progress in understanding the disease and the development of new therapeutic strategies, asthma-related morbidity and mortality continued to increase over the last decades. Although essential, providing optimal asthma management in children encounters a number of challenges, from establishing appropriate diagnosis to early treatment with adjustment and monitoring [1,2,3,4].

In recent years, asthma research has focused on severe forms of the disease, achieving encouraging results in the field of biological therapies. However, severe asthma accounts for no more than 2% of all cases of bronchial asthma in the pediatric population and most exacerbations are seen in patients with mild and moderate forms of asthma [5]. Non-severe asthma therefore remains a major health problem, both for patients and their caregivers and for the specialists involved in their care. Recent evidence suggests important benefits from the use of anti-inflammatory medication even for mild forms of the disease that previously only benefited from bronchodilator therapy when needed. Bronchodilator medication has also been shown to produce positive outcomes in treatment combined with inhaled corticosteroids. “Single maintenance and reliever therapy” (SMART or MART) is a new treatment option in moderate disease, and it has been successfully incorporated into current guidelines. Both Global Initiative for Asthma (GINA) and the National Asthma Education and Prevention Program Coordinating Committee (NAEPP) recommend the use of a single inhaler containing the combination of an inhaled corticosteroid (ICS) and formoterol, a specific fast-acting bronchodilator (FABA), for both maintenance and quick relief therapy in steps 3 and 4 of asthma management [6,7,8]. As for uncontrolled, difficult-to-treat asthma and severe asthma, an appropriate evaluation is required in order to assure the right diagnosis and management. Before treatment step up, it is important to exclude an incorrect diagnosis, a poor adherence to treatment, incorrect inhaler technique or the presence of comorbidities. Treating associated comorbidities improves asthma outcomes as well. For example, children with asthma must be evaluated for gastroesophageal reflux in order to treat, as it can help the treatment of asthma or even solve some resistant to standard treatment cases [9].

### 1.1. Definition

Despite multiple attempts to reach a consensus on the definition of asthma, at least 60 different definitions of pediatric bronchial asthma have been identified in 122 published studies. Although some of those may appear almost identical, the impact they have on the clinical management of the disease is substantially different [4]. The GINA 2022 guidelines define asthma as a heterogeneous condition, typically characterized by chronic airway inflammation and the presence of respiratory symptoms, such as wheezing, dyspnea, chest tightness and cough, the intensity of which may vary over time, associated with variable expiratory airflow limitation, which may become persistent over time [6].

### 1.2. Diagnosis

Correct diagnosis of the disease is an important first step in its management. In many European countries, diagnosis is still based on history and clinical examination, without additional investigations. As a result, several studies in Europe and North America have shown an increased rate of misdiagnosis with asthma being both under- and over diagnosed [10]. Overdiagnosis resulted in misuse of medication, especially corticosteroids, with possible associated side effects, increased medical bill costs and in some cases delays in establishing the correct diagnosis. On the other hand, under-diagnosis and inappropriate treatment of cases led to decreased quality of life, increased morbidity and mortality, especially in resource-limited healthcare units [1,11].

### 1.3. Asthma Phenotypes, Genotypes and Biomarkers

Type 2 (T2) inflammation represents the keystone in asthma pathogenesis. Childhood asthma is known to be frequently characterized by atopy and a T2 high endotype, although current evidence sustains the existence of another T2 low endotype, with different underlying airway immune-mediated inflammation. Type 2-high asthma is described by eosinophilic inflammation, promoted by T2 cytokines as IL-4, IL-5, and IL-13, with IL-3, IL-9, IL-10, and GM-CSF included in broader classification [12,13]. The secretion of interleukins is triggered by an allergen, germ or pollutant which comes in contact with bronchial epithelial cells and followed by the recruitment of eosinophils, mastocytes and basophils in the respiratory airway as well as an Ig E production [14]. T2-high asthma is also associated with increased CD3+, CD4+, and CD8+ lymphocytes and altered dendritic cells. It denotes a good response to corticosteroid (CS) treatment and due to its biomarker identification, benefits from novel biological therapies, especially the severe cases of asthma [13,15].

T2-low asthma is represented either by a neutrophilic or a paucigranulocytic inflammatory pattern and it is sustained by IL-8, IL-17A, IL-2 and other T cell-related cytokines, including epithelial cell-derived cytokines. It is a rare endotype, found in patients with severe disease forms. It usually displays an insensitivity to corticotherapy, associated with a corticosteroid resistance [12,13].

These endotypes are characterized by the presence of some specific biological molecules. A biomarker is a measurable biological indicator which can estimate, in objective approach, the status of health or the disease progression, predict the response to therapy and monitor disease evolution [16]. A valid biomarker needs to be available for the clinicians and be reliable. It can be measured in the sputum, bronchoalveolar lavage (BAL), exhaled breath condensate (EBC), bronchial biopsy, urine and blood. In current practice, the biomarkers that are frequently used are associated with T2 inflammation: blood or sputum eosinophils, serum Ig E and fractional exhaled nitric oxide (FeNO). 

Eosinophils, with a blood count cutoff value of 300 cells/microL or a sputum cell count above 2–3% of the total cell count, are usually used to monitor severe forms of the disease [15,16]. Eosinophil blood count corresponds to asthma severity and airway hyperreactivity (AHR) in pediatric patients and when associated with FeNO, illustrates a good inhaled corticosteroid (ICS) response. Different cutoff values of eosinophilia can be successfully used as a reliable reference, allowing practitioners to identify individuals with specific features of severe asthma that can benefit from varieties of available biological therapies [17]. As for sputum eosinophilia, it does not always correlate with peripheral blood film values. A high number of eosinophils in sputum stands for atopy, AHR, asthma, and has the capacity to predict future severe exacerbations, as well as the response to treatment [12,15].

Total and allergen-specific IgE levels reflect the severity of clinical symptoms, being a hallmark for atopic status in asthma patients. Total IgE is linked to the risk of developing asthma later in life, for infants with viral-induced wheezing, while asthma severity will follow allergen-specific IgE levels [12,16].

FeNO is a noninvasive biomarker measured in exhaled breath. Allergen exposure leads to high levels of nitric oxide (NO), making FeNO a marker for eosinophilic inflammation of the airways. It corresponds to AHR, serum IgE levels and blood eosinophils, and the response to ICS administration. It can be assessed as a predictor of control loss [12,15].

Serum periostin, despite its proven role in diagnosis and correlation to severity of clinical symptoms and prediction of treatment response in adults, in the pediatric department its position is still debated [15].

### 1.4. Classification of Asthma Severity

Currently, asthma severity is assessed retrospectively after the treatment over a certain period of time, considering the symptom control and exacerbations. Thus, mild asthma is defined as a disease condition which is well controlled on low dose ICS or as-needed-only ICS-formoterol. Moderate forms are those controlled with low- or moderate-dose ICS in combination with long-acting beta 2-agonists (LABA), and severe forms remain uncontrolled, despite high-dose ICS combined with LABA or other specific drugs, with good patient adherence, correct administration technique and adequate treatment of comorbidities [6,18,19,20].

## 2. Asthma Medication

Drugs in asthma include fast-acting rescue medication and long-term control medication. Quick relief medicines have the role of relieving acute asthma attacks as well as suppressing exercise-induced bronchoconstriction (EIB) symptoms. This category encompasses SABA, anticholinergics (used only for severe exacerbations), and systemic CS. Long-term controllers include ICS, LABA, long-acting anticholinergics, combination ICS and LABA, methylxanthines, leukotriene agonist receptor (LTRA) and monoclonal antibodies [6,21].

Beta2-adrenergic agonist agents relieve reversible bronchospasm by relaxing the smooth muscles of the airway. They are used for both acute and long-term asthma therapy. SABAs are the most effective bronchodilator drug, with rapid onset of action and the first choice of reliever drug for many years, in both children and adults [3]. Recently, as described above, due to their adverse reaction, their use is conditioned by the concomitant administration of ICS, with several exceptions. They are available as an inhale administration or nebulizer, intravenous or oral use tablets and oral suspensions [6]. Their effectiveness is based on the stimulation of beta2-adrenergic receptors, leading to bronchodilation, the increase in mucociliary clearance, the decrease in vascular permeability, inhibition of nervous fibers and modulation of mast cell mediator release. Salbutamol is in the class of most-used drug, which delivers instant relief estimated 1 min after an administration via spray/multidose inhaler with 4 to 6 h of extended effect. SABA therapy has a cumulative effect, small, repeated amounts having an increased action compared to a single administration of a higher dose [3,22].

LABAs have similar bronchodilator action as SABAs, except a longer duration of effect. As for the two commonly used LABAs, there is a more rapid onset of action of formoterol (2–5 min) when compared to salmeterol (15–30 min) [23]. Another bronchodilator drug, Vilanterol, is an inhaled ultra-long-acting beta-2 agonist (ULABA) which is recommended in combination with the ICS fluticasone furoate, as an alternative treatment for children ≥12 years old with moderate persistent forms of asthma [6]. Once daily combined ICS-ULABA led to improvement in asthma control in a group of adolescents with prior poor adherence to inhaled therapy [24]. A recent study reported that fluticasone furoate–vilanterol use is more cost effective than the usual combination of fluticasone propionate–salmeterol [25]. Further research of vilanterol use in pediatric patients is required.

They must never be used as monotherapy, but always be administrated in association with ICS, due to FDA warnings on occurrences of severe asthma exacerbations resulting in the demise of some patients. When associated with ICS or anticholinergic drugs, no similar adverse reaction was reported. Salmeterol and formoterol are frequently used in current practice, in different combinations with ICS [6,22].

Anticholinergic agents produce bronchodilation through the reduction of vagal tone of the airways, blocking the bronchoconstriction effect when in contact with allergens. They are used in acute symptoms, for patients not able to tolerate inhaled sympathomimetic or for the prevention of night symptoms (it inhibits the physiological nocturnal vagal hypertonia) [3]. Shorter-acting medicines typically used include ipratropium bromide, and longer-acting anticholinergic medicines include tiotropium, aclidinium, glycopyrronium, and umeclidinium. These medications have the ability of binding and blocking neural signals from parasympathetic muscarinic receptors M1, M2, and M3. M3 receptors are located along the airway, on smooth muscle cells, mucosal glands and vascular endothelium and their inhibition leads to many benefits in asthma treatment [6].

Tiotropium is a long-acting muscarinic agonist (LAMA) agent. It targets M3-receptors located on smooth muscle, leading to bronchodilation. It is the only drug in this category approved for use in uncontrolled asthma cases of patients aged 6 years and older. It is used as a long-term maintenance treatment, given once daily [6]. It has a moderate effect on improving lung function and reducing exacerbation rates. Current literature described that tiotropium has the ability to reduce the risk of exacerbation in severe persistent asthma patients aged 1–5 years, with the similar adverse effects of actual standard treatment. However, their introduction as an alternative option of therapy in children younger than 5 years needs further investigation [2,20].

Ipratropium is a short-acting anticholinergic agent, chemically similar to atropine. Together with SABA it is recommended in moderate–severe asthma attacks, within the first hour of treatment, even in patients 5 years or younger [6]. Those two combined have a greater bronchodilation effect than either drug administrated in monotherapy. Their use achieves a decrease in hospital admissions rates, improves lung function and reduces risk of tremor and nausea, compared to beta2-agonists monotherapy [22].

Steroids are the most powerful anti-inflammatory therapy in asthma, as they interfere with arachidonic acid metabolism, leukotriene and prostaglandins synthesis, histamine release and inflammatory pathways. They amplify the response of beta2-adrenergic receptors from the airway to bronchodilators, they decrease mucus secretion and serum IgE levels. Administration can be made parenteral, oral or through inhalers/nebulizers. Most commonly used oral corticosteroids (OCS) in asthma treatment include prednisone, prednisolone, methylprednisolone and dexamethasone. They are used for short periods of time to achieve swift control over acute exacerbation of the disease [22]. Given in the emergency department they have the capacity of reducing the risk of hospitalization, although their effect on hospitalization risk if administrated in the outpatient setting is still debated. When utilized for a long time, their aim is to prevent exacerbation in severe persistent asthma as well as maintaining suppression, control and reverse the inflammatory process. Several adverse effects are associated with their use, both local and systemic [3,6].

Intravenous CS are recommended in special situations, such as “status asmaticus”, in order to achieve rapid control and symptom remission, without adverse outcomes. The intravenous use is also recommended when the oral administration is not well tolerated or when there is no response to initial treatment [22].

ICS are the most efficient treatment of asthma, as a part of GINA step 2, 3 and 4. Their use early in asthma progression improves disease control, lung function and prevents airway remodeling phenomena. The side effects are barely local, as they are topically active and poorly absorbed into systemic circulation. The association with LTRA may benefit from the decrease in ICS doses required to gain control of severe asthma. Fluticasone, beclomethasone and budesonide are the most used ICS in clinical practices [6].

Nonselective phosphodiesterase enzyme inhibitors are used for symptom prevention and long-term control, with a particular effect on nocturnal symptoms. Theophylline, available in short- and long-acting products, has a bronchodilator effect through its antagonist action on adenosine receptors and through its nonselective inhibitory action on phosphodiesterase enzyme. The utilization is limited by its pharmacokinetic particularities: great interindividual variability, metabolic interferences with several factors, i.e., fever, concomitant use of macrolides, cimetidine or antifungal drugs [22].

Mast cell stabilizers (cromolyn sodium) are anti-inflammatory drugs, with the capabilities to stabilize the mast cell membrane, ceasing the release of inflammation mediators. In acute episodes, they prevent the bronchospasm induced by effort, cold air or allergen exposure. They have no intrinsic anti-inflammatory, antihistamine, or vasoconstrictive effects [21].

LTRA represent an anti-inflammatory option for long-term treatment in children. There are two categories of LTRA, depending on their underlying mechanism: the inhibition of 5-lipoxygenase and the reduction of bronchoconstrictor effect, leukotriene synthesis, LTC 4 and LTD 4. Montelukast is the most recommended drug used in monotherapy for children with viral-induced asthma, effort induced disease or in patients associating allergic rhinitis or atopic dermatitis. In step 3 or 4, it can associate with ICS in order to decrease ICS doses. Side effects include emotional, behavior and sleep disorders, frequently observed seen in clinical practice [6].

Monoclonal antibodies used in asthma therapy are human monoclonal antibodies (MAbs), derived from a B-lymphocyte clone. They target cytokines with a specific role in inducing and maintaining eosinophilic inflammation, with inhibitory effects on IgE binding and competitive inhibition of allergen binding and IgE complex formation. Monoclonal antibodies are recommended for certain types of severe asthma with a specific biomarker profile. Most of them aim for the Th2 high asthma profile, but recently a biological agent targeting Th2 low asthma endotype has been approved for pediatric use [15,26].

Omalizumab, the first biological agent used in asthma treatment, is an anti-IgE humanized murine monoclonal antibody. It has the ability of binding to the Fc region of IgE, not allowing free IgE to bind to IgE receptors. It decreases the downstream inflammatory cascade and obtains an attenuated allergic response. It is currently approved for usage in adults and children aged 6 and older, with moderate and severe asthma. The administration is subcutaneous, made every 2 to 4 weeks, with a dosage dependent on body weight and serum total IgE levels, determined before the beginning of the therapy. It has good results in reducing ICS dosage, reducing exacerbation of the disease and improving the quality of life [15,26,27].

Mepolizumab is an anti-IL 5 humanized murine monoclonal antibody approved for subcutaneous use in children 6 years old and older. It is administrated every 4 weeks, in a 40 mg or 100 mg dose, depending on age category. It reduces exacerbation of the disease, improves the quality of life and has a steroid sparing effect [27,28].

Benralizumab is a humanized monoclonal antibody that binds to IL-5 receptor alpha subunit of eosinophils and basophils, leading to target cell apoptosis. Recommendations are based on subcutaneous use in children aged 12 years and older. The therapeutic scheme includes a 30 mg dose given every 4 weeks for three doses, followed by every 8 weeks. It has the ability of reducing exacerbation rates and has a sparing effect on corticosteroid use in patients whose asthma is not controlled by combination of medium- to high-dose ICS and LABA. It also improves lung function [26,27,28,29,30]. Its use in children is approved exclusively in the USA. Currently, Europe recommends benralizumab treatment only in adults [31].

Dupilumab is a monoclonal antibody anti-IL 4 receptor alpha, aiming at IL-4 and IL-13 pathways. The usage is approved for children aged 12 years and older, diagnosed with a form of moderate to severe persistent asthma associated. For subcutaneous use, the recommended dose is 200 mg or 300 mg every 2 weeks. When associated to severe asthma with an OCS dependence, a 300 mg dose is recommended, given at the same frequency. Patients aged 6–11 years may also benefit from dupilumab, with dose and frequency weight dependent. The treatment yields a significant reduction in asthma exacerbations in patients treated with ICS-LABA, resulting in increased quality of life, symptom control and lung function improvement [27]. Dupilumab, mepolizumab, and benralizumab can be self-administrated at home, using prefilled injectors [15].

Tezepelumab is a monoclonal antibody targeting thymus stromal lymphopoietin (TSLP), an epithelial-derived cytokine associated with asthma exacerbation. It is the first biological agent approved in treating both types of severe asthma, regardless of biomarkers and allergic status [26]. Starting with GINA 2022, it is accepted for patients 12 years and older as step 5 add-on therapy, in subcutaneous use, with 210 mg given every 4 weeks [6]. Tezepelumab treatment was associated with a reduction in severe exacerbation rates, an improved quality of life, lung function and symptom control, independent of allergic status. Clinical results seem to be in a direct relationship with blood eosinophils and FeNO levels [26,27].

## 3. Pharmacological Treatment of Asthma

Currently, the treatment of the pediatric patient with asthma comprises two stages: initial treatment, instituted at the patient’s first assessment, and subsequent treatment guided by the course of the disease. The first treatment regimen is chosen according to the presence and frequency of day/night symptoms, limitation of physical activity and the risk of exacerbations. Subsequently, it is based on the progress of the patient who has been put under a treatment program, and asthma may be classified as controlled, partially controlled or uncontrolled [6,32].

Historically, the treatment of asthma has varied, from chickweed cigarettes, a natural anticholinergic, used until the 1990s, to inhaled adrenaline, used in the first half of the 20th century. This was the first drug administered using a pressurized Metered Dose Inhaler (pMDI) and was replaced a decade later by selective beta2-agonists, such as salbutamol. As for ICS, their benefit and safety have been demonstrated since the early 1970s and they have since become part of standard therapy for bronchial asthma. Over a period of time, it has been shown that the anti-inflammatory effect of ICS is limited and that combining them with a second drug has shown to be significantly more effective than the increase in ICS doses in the chronic treatment of asthma. Thus, for some time the treatment guidelines have been introduced, based on the use of SABA as a crisis medication and ICS as a maintenance drug of choice—a chronic medication [33,34].

Long-term use of SABA has been associated over the years with a number of adverse reactions, such as reduced ß-receptor numbers, reactivation of bronchial hyper-reactivity (rebound phenomenon), reduced bronchodilator response, and increased allergic response and eosinophilic airway inflammation [35,36]. An unfavorable clinical outcome has been observed with the daily use of more than three vials of SABA/year, along with an increased risk of referral to the emergency room [37,38,39]. An increased risk of death has also been reported after consumption of 12 vials/year [39,40].

Studies suggest that these SABAs are used preferentially by patients over the usual ICS or ICS/LABA combinations, thereby masking a worsening clinical picture [40]. Since 2019 the GINA guidelines, based on a stepwise, progressive approach to asthma severity, which bring significant updates to the management of mild forms of bronchial asthma, have abandoned the monotherapy use of SABA as needed in adults and children older than 12 [41].

GINA 2021 comes with a new bronchial asthma therapeutic regimen, which, depending on the chosen controller medication, divides treatment options into two strategies, a preferred one and an alternative one, each with five steps corresponding to the patient’s symptoms. The recommendations remained unchanged in GINA 2022. Thus, strategy 1, preferably for patients aged 12 years and older, comprises low-dose ICS-formoterol as control therapy at all GINA steps of severity: only as needed in asthma steps 1 and 2 and with daily maintenance dose ICS-formoterol (maintenance and control therapy—MART) in steps 3–5. Strategy 2 (alternative) describes for step 1 the administration of SABA on an as-needed basis together with a low dose of ICS at the same time or immediately after SABA administration. Steps 2–5 recommend maintenance treatment with ICS-LABA and, whenever needed, SABA [6].

In the 2022 GINA report, children aged 6–11 years with symptoms occurring less than twice a month (stage 1) are recommended to take ICS whenever SABA is given as crisis therapy. When symptoms occur at least twice a month—but less frequently than daily (step 2)—ICS is recommended daily as maintenance therapy and SABA whenever needed. In steps 3 and 4 of severity, MART-administration of ICS-formoterol in low or very low doses is recommended, preferably. Finally, the GINA 2022 report recommends that in any severity step of bronchial asthma, crisis treatment should include SABA or ICS-formoterol if needed [6].

**Figure 1:**
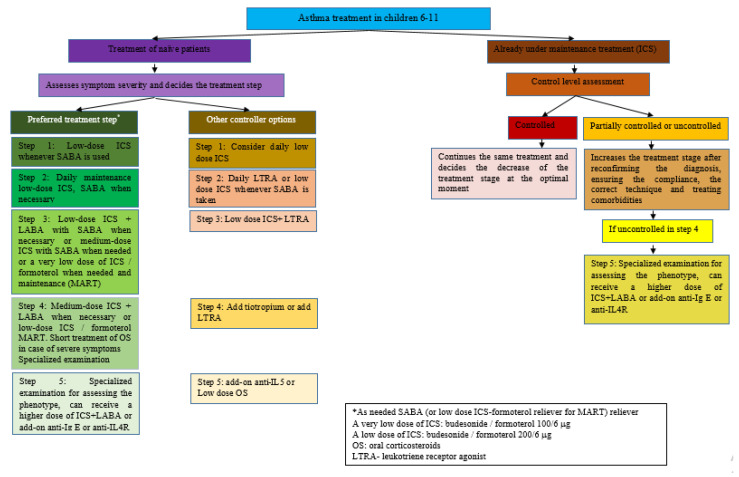
Asthma treatment based on GINA 2022 recommendations for children aged 6–11 [6] and adapted from Recent Advances in Long-Term Management of Asthma (published online ahead of print, 20 January 2022). Indian J Pediatr. 2022 [42].

**Figure 2:**
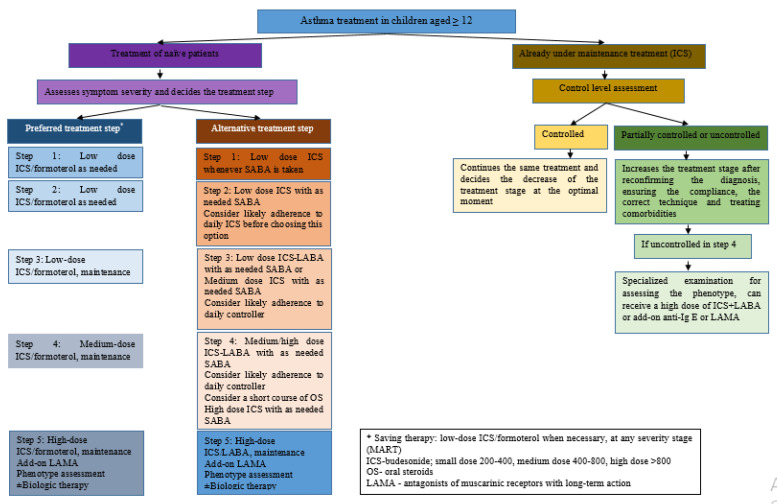
Asthma treatment based on GINA 2022 recommendations for children aged ≥12 and adapted from Recent Advances in Long-Term Management of Asthma (published online ahead of print, 20 January 2022). Indian J Pediatr. 2022 [42].

The GINA 2022 recommendations for the 5–11 years and ≥12 years of age categories are summarized in Figure 1 and Figure 2, respectively.

To sum up the SMART approach, it is recommended as a preferred treatment for both adolescents 12 years old or older and children 5–11 years old, with moderate disease forms. It significantly reduces the risk for severe exacerbations compared with maintenance ICS or ICS-LABA regimens with a SABA reliever [42,43]. Step 3 indicates one low dose of budesonide/formoterol given once or twice daily for adolescents aged ≥12 years old and one very low dose of budesonide/formoterol given once daily for children aged 5–11 years old, as maintenance therapy, in association with a dose of budesonide/formoterol as reliever therapy, given one inhalation as needed. Step 4 recommends two medium doses of budesonide/formoterol given twice daily for adolescents aged ≥12 years old and one low dose of budesonide/formoterol given twice daily for children aged 5–11 years old, as maintenance therapy, in association with a dose of budesonide/formoterol as reliever therapy, given one inhalation as needed [6].

For initial wheezing episodes in children younger than 5 years, SABA is recommended as needed. Patients in this age group to be treated according to stage 2 will receive low-dose ICS or LTRA as a secondary option to maintenance treatment, stage 3 will receive double-dose ICS or low-dose ICS and LTRA, and stage 4 patients require specialist assessment for targeted treatment of comorbidities; regardless of severity stage, patients will receive SABA anytime as needed [6].

Although most European countries use the GINA guidelines, globally there is a lack of uniformity in asthma management protocols due partly to national regulations on the use of certain drugs, but also to their low availability [22,44]

The National Asthma Education and Prevention Program (NAEPP) 2020 envisions, like GINA, a stepwise approach to asthma in severity steps, offering two treatment strategies, one preferred and one alternative. Both guidelines recommend ICS/formoterol combination as maintenance but also control medication as a treatment option starting with step 3 in patients ≥5 years of age. The NAEPP goes further and recommends ICS/LABA also under 5 years of age as maintenance treatment for forms corresponding to stage 4 and above, while GINA restricts the use of this combination to patients aged ≥ 5 years. Differences also occur in the treatment of mild forms of asthma, where the NAEPP still recommends SABA monotherapy in all age groups [45]. Nor has the Canadian Thoracic Society abandoned the use of SABA monotherapy as a treatment for mild asthma, but it remains the first-line treatment in patients with bronchial asthma well controlled by SABA when needed and without risk factors, regardless of age. The ICS/formoterol combination is an option for treatment of all forms of asthma only in patients aged 12 years and older [46]. The option to administer ICS at each SABA administration which exists in the GINA recommendations is not approved in Canada and is available only for adults with a high risk of exacerbations [46].

### 3.1. Uncontrolled, Difficult-to-Treat Asthma and Severe Asthma

GINA defines uncontrolled asthma as a form of asthma in which poor symptom control is achieved (frequent presence of symptoms or frequent use of crisis medication, limitation of physical activity and night-time awakenings due to asthma) or which is characterized by exacerbations requiring either oral corticosteroids (≥2/year) or hospitalization (≥1/year).

Difficult-to-treat asthma is uncontrolled asthma despite a medium or high dose of ICS and of a second maintenance drug or oral corticosteroid as maintenance treatment (GINA steps 4 and 5). It may be the result of incorrect diagnosis, poor adherence to treatment, incorrect inhaler technique or the presence of comorbidities.

Severe asthma is defined as asthma that remains uncontrolled despite optimized treatment with high dose ICS-LABA, or that requires high dose ICS-LABA to prevent it from becoming uncontrolled LABA in patients with good adherence, correct inhaler technique and optimal treatment of comorbidities [6].

### 3.2. Management of Severe Asthma

**Figure 3:**
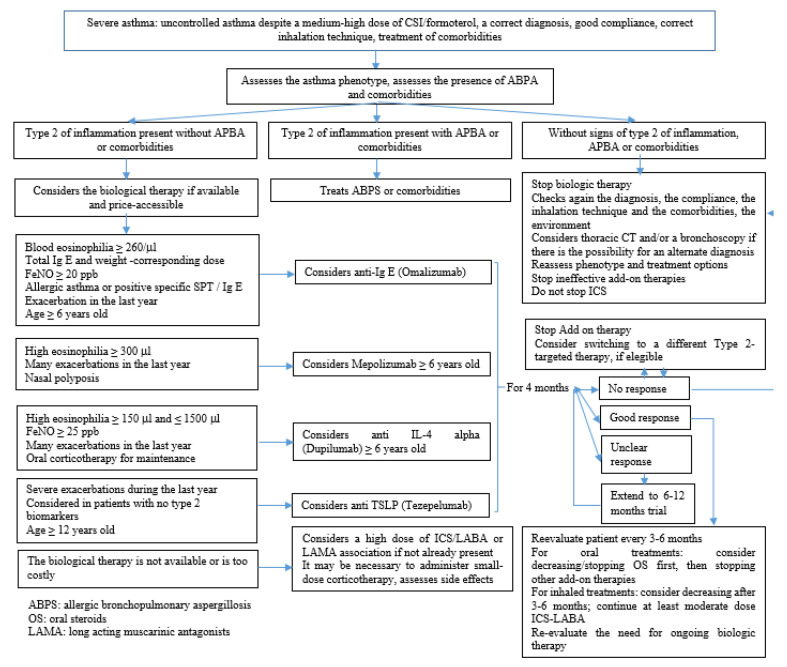
Management of severe forms of asthma adapted to GINA 2022 recommendations and from Recent Advances in Long-Term Management of Asthma (published online ahead of print, 20 January 2022). Indian J Pediatr. 2022 [42].

Severe asthma is a complex and heterogeneous condition, and the identification, as precisely as possible, of phenotypes (clinical panels) caused by various endotypes (distinct physio-pathological and immunological mechanisms) is important for choosing the optimal therapy for each patient [46,47]. A severe asthma treatment algorithm is shown in Figure 3.

### 3.3. Management of Exacerbations of Bronchial Asthma

Exacerbations are acute or subacute episodes of worsening of respiratory symptoms and lung function (measurable by a decrease in peak expiratory volume in the first second—FEV1, and peak expiratory flow—PEF) compared with the patient’s usual condition. Objective measurement of PEF is used in the classification of asthma severity in children, but this is difficult in patients with deteriorating clinical status or those of very young ages [48,49].

Acute exacerbations of bronchial asthma are the leading cause of hospitalization of pediatric patients in the United States, with an average length of hospitalization of 1.4 days. Most patients with bronchial asthma are admitted for less than 1 day and can be managed effectively in outpatient settings if time and resources are available [50]. A recent meta-analysis revealed that intensive care unit admission for an asthma exacerbation is associated with an increased risk of future admission [51].

Analysis of current protocols used in the management of acute exacerbations demonstrates variability in the treatments applied in the emergency room. However, the common goal of treating an acute episode is to prevent progression to acute respiratory failure and to treat respiratory symptoms by combating bronchoconstriction and reducing lung inflammation [3,50].

Determination of attack severity is mainly based on clinical criteria (respiratory frequency, presence of wheezing and existence of sternocleidomastoid muscle retraction). Although there is no well-validated clinical scale, the lung score (Table 1) is simple to apply at any age [3,14].

**Figure 4:**
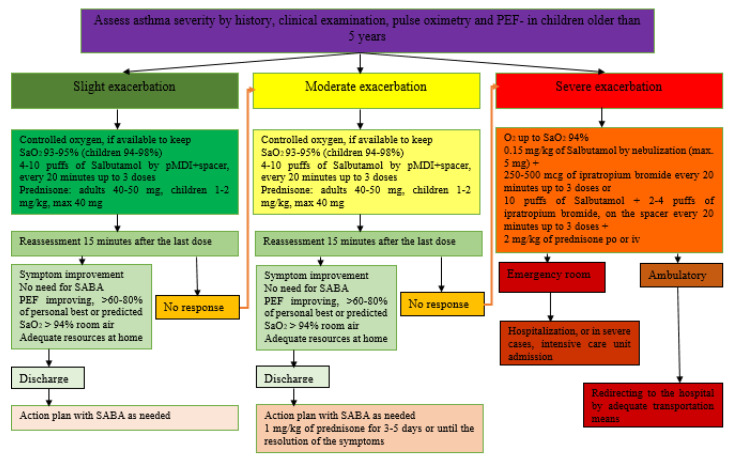
Therapeutic management of acute exacerbations adapted from the Spanish asthma management guide GEMA 5.0 [3] and GINA 2022 [6].

The severity of the episode can be easily assessed by associating the severity of symptoms with the blood oxygen saturation (SaO_2_) illustrated in Table 2. Figure 4 represents the therapeutic protocol for asthma exacerbations proposed by the Spanish Pediatric Consensus and GEMA5.0 guidelines according to their severity.

The basic medications used in the treatment of exacerbations include bronchodilators, oxygen therapy and glucocorticosteroids [3]. Thus, correction of significant hypoxemia will be performed with oxygen supplementation—severe cases with alveolar hypoventilation will require mechanically assisted ventilation; rapid reversal of airflow obstruction will be performed by administration of an inhaled beta2-agonist [6,52]; their intravenous administration in patients with severe exacerbations is not recommended by GINA. Early administration of systemic corticosteroids is beneficial in patients with bronchial asthma who do not respond promptly and completely to inhaled beta2-agonists [53]. In terms of reducing the risk of recurrence of symptoms, a short course of systemic corticosteroids is cited as useful [20].

GINA 2022 does not routinely recommend performing a chest X-ray or administering antibiotics in exacerbations. The decision to hospitalize the patient will be made based on the patient’s clinical status, lung function, response to treatment, history of exacerbations, social support, and ability to comply with therapeutic recommendations at home [6,20,53,54,55].

## 4. Inhaled Corticotherapy Doses Used for Pediatric Patients

In medical practice, the medication, the inhalation device and the therapeutic dose must be chosen for each patient, depending on the assessment of the control over the disease, of the present risk factors, of the patient’s preference, and some practical considerations (cost, ability to use the device, treatment compliance). The monitoring of the treatment response and the precise adjustment of the doses are important. When good symptom control is reached and maintained for 3 months, the ICS dose should be carefully managed up to the lowest value that can maintain the control of the symptoms and minimize the side effects. Low ICS doses bring considerable clinical benefits for most patients. Nevertheless, the response to ICS varies from one patient to another, as some of them require medium doses when their asthma form remains uncontrolled or presents exacerbations despite good treatment compliance or a correct technique for administering a low dose of ICS (in association with LABA or not). Low doses of ICS (in association with LABA or not) are necessary for a limited number of patients, and their long-term administration is associated with a high risk of side effects, which must be balanced with their potential benefit, when a therapeutic decision is taken [6]. The total doses of ICS recommended by GINA for treating asthma in children and adolescents, according to the studies and information regarding each product, are presented below (Table 3, Table 4 and Table 5).

The numbers shown in Table 5 are suggestions for low total doses of daily administered ICS treatment for children aged 5 and below, recommended based on current studies and product information. The shown doses are the lowest approved doses, safe and efficient for the targeted age group [6].

## 5. Vaccination in the Asthmatic Patient

Early immunization has long been regarded as a promoter of asthma development by stimulating a Th2-type immune response or by decreasing microbial stimulation and tilting the balance between Th1 and Th2; however, no association has been identified between the administration of acellular diphtheria–tetanus–pertussis vaccine, polio vaccine or measles–mumps–rubella vaccine and increased risk of developing bronchial asthma. Hooker et al. analyzed the outcomes of vaccinated versus unvaccinated children during their first years of life. Children at least 3 years of age were included. Higher ORs were observed within the vaccinated versus unvaccinated group for asthma. However, since the study only allowed for the calculation of unadjusted observational association, there is a need for additional investigation [56]. A recent meta-analysis studied the relationship between childhood vaccination and allergic disease. BCG vaccine, measles/measles, mumps and rubella (MMR) vaccine, acellular pertussis and whole cell pertussis vaccine were included. The results did not support the association of early life vaccination and an increased risk of allergic disease. Measles vaccine however could be linked to a reduced risk of eczema, but further studies are required [57]. On the other hand, a Danish study evaluating the risk of childhood asthma after live MMR vaccine and non-live diphtheria–tetanus–acellular-pertussis–inactivated-polio–Haemophilus influenzae type b (DTaP-IPV-Hib) vaccine concluded that MMR may have a protective effect against childhood asthma for boys [58]. The association between asthma and vaccination against hepatitis B virus (HBV) and *Hemophilus influenzae* type B is weak [59,60,61,62,63]. A case–control study on the risk of Hib combination vaccination and history of pneumonia for asthma symptoms among 5-year-old Indian children concluded that Hib combination vaccination was a protective factor against wheezing in 0-year-old children. However, the effects of vaccination might have attenuated at the ages of 1–4 years, because no booster dose was administered. The addition of a booster dose might further decrease the prevalence of asthma and wheezing [64].

Regarding immunity of asthma patients acquired from immunizations, studies find maintenance of protective antibody titers 3 years after completion of the usual vaccination regimen. However, this immunity tends to be lower in patients with moderate forms of asthma after vaccination against some viruses (inactivated HBV and attenuated anti-rubella vaccines) [63].

Asthma patients, particularly young children, are at up to six times greater risk of developing invasive pneumococcal infections, the U.S. Advisory Committee on Immunization Practices (ACIP) recommends pneumococcal vaccination for pediatric patients with bronchial asthma on high-dose corticosteroid therapy [65].

The influenza virus is responsible for significant morbidity and mortality in the general population, contributing to some exacerbations in patients with bronchial asthma. An adult study estimated that, on average, current or previous influenza vaccination of people with asthma prevented almost half of influenza cases. Influenza vaccination effects were similar for patients with and without asthma [66]. As for the safety of Live Attenuated Influenza Vaccine (LAIV) and Inactivated Influenza Vaccine (IIV) in children with asthma, Ray et al. found no increased risk of asthma exacerbation following LAIV or IIV [67]. GINA recommends that patients with moderately severe forms of asthma should receive an influenza vaccine annually or when the general population is advised to do so [6]. Despite recommendations for children with asthma to receive the influenza vaccine, vaccination rates in this population remains low [68,69].

Currently, considering both risks and benefits, GINA recommends COVID-19 vaccination for people with asthma aged ≥5 years. A 14-day interval should be considered between administration of the flu vaccine and COVID-19 vaccine, due to a lack of studies on the safety and efficacy of the latter when administered concurrently with other vaccines, says the Centers for Disease Control and Prevention. In patients with severe asthma on biologic therapy, the vaccine and the drug should not be administered on the same day to allow for the possibility of distinguishing adverse reactions when they occur [6].

## 6. Conclusions

Asthma is a broad term which has been widely used for a cluster of respiratory conditions with similar symptoms but arise from different etiologies, endotypes and phenotypes. Taking control of this disease requires an extensive regimen following various treatment guidelines introduced by world-leading organizations aiming at treating mild to severe forms of disease conditions. It is evident that we still need to put in extensive amounts of research and development works to support the validity of current recommendations, which are in the process of globally aligned standards and practices. We strongly believe that phenotype/endotype based personalized therapy should be the right path to the discoveries of new therapies, with the hope of achieving a better outcome of disease control.

## Figures and Tables

**Table 1:** Lung score for clinical assessment of asthma exacerbation in children according to the Spanish Guidelines for Asthma Management GEMA 5.0 [3].

Score *	Respiratory Rate		Wheezing
	<6 Years	≥6 Years	
0	<30	<20	Without
1	31–35	21–35	At end of breath
2	46–60	36–50	Throughout exhalation (with stethoscope)
3	>60	>50	Inhaling and exhaling (without stethoscope) **

* Score from 0 to 3 for each section (minimum 0, maximum 9). ** If there is no wheezing and sternocleidomastoid muscle use is increased, the section corresponding to wheezing will receive 3 points.

**Table 2:** Global assessment of asthma exacerbation in children, including pulmonary score and oxygen saturation based on the Spanish asthma management guide GEMA 5.0 [3].

	Lung Score	SaO_2_
Mild	0–3	>94%
Moderate	4–6	91–94%
Severe	7–9	<91%

In case of a discrepancy between clinical score and oxygen saturation, the most severe value will be considered.

**Table 3:** Daily total dose of ICS recommended to adolescents aged ≥12 for asthma treatment based on GINA 2022 [6].

Adolescents ≥12 Years Old			
ICS Type	Daily Total Dose of ICS (mcg)		
	Low	Medium	High
**Beclomethasone dipropionate** (pMDI, standard particles, HFA)	200–500	>500–1000	>1000
**Beclomethasone dipropionate** (DPI or pMDI, extra fine particles, HFA)	100–200	>200–400	>400
**Budesonide** (DPI or pMDI, standard particles, HFA)	200–400	>400–800	>800
**Ciclesonide** (pMDI, extra fine particles, HFA)	800–160	>160–320	>320
**Fluticasone furoate** (DPI)	100		200
**Fluticasone propionate** (DPI)	100–250	>250–500	>500
**Fluticasone proprionate** (pMDI, standard particles, HFA)	100–250	>250–500	>500
**Mometasone furoate** (DPI)	It depends on the used device—see product information		
**Mometasone furoate** (pMDI, standard particles, HFA)	200–400		>400

DPI: dry powder inhaler; HFA: hydrofluoroalkane propellant; ICS: inhaler corticosteroid; pMID: pressurized inhaler with fixed dose.

**Table 4:** Daily total dose of ICS recommended to children aged 6–11 for asthma treatment based on GINA 2022 [6].

Children Aged 6–11			
ICS Type	Daily Total Dose of ICS (mcg)		
	Low	Medium	High
**Beclomethasone dipropionate** (pMDI, standard particles, HFA)	100–200	>200–400	>400
**Beclomethasone dipropionate** (DPI or pMDI, extra fine particles, HFA)	50–100	>100–200	>200
**Budesonide** (DPI)	100–200	>200–400	>400
**Budesonide** (nebulization)	250–500	>500–1000	>1000
**Ciclesonide** (pMDI, extrafine* particles, HFA)	80	>80–160	>160
**Fluticasone furoate** (DPI)	50		Without indications
**Fluticasone propionate** (DPI)	50–100	>100–200	>200
**Fluticasone propionate** (pMDI, standard particles, HFA)	50–100	>100–200	>200
**Mometasone furoate** (pMDI, standard particles, HFA)	100		200

DPI: dry powder inhaler; HFA: hydrofluoroalkane propellant; ICS: inhaler corticosteroid; pMID: pressurized inhaler with fixed dose.

**Table 5:** Daily total dose of ICS recommended to adolescents aged ≥5 for asthma treatment based on GINA 2022 [6].

Children Aged ≤5	
ICS Type	Daily Total Low Dose of ICS (mcg)
**Beclomethasone dipropionate** (pMDI, standard particles, HFA)	100 (≥5 years old)
**Beclomethasone dipropionate** (DPI or pMDI, extra fine particles, HFA)	50 (≥5 years old)
**Budesonide** (nebulization)	500 (≥5 years old)
**Fluticasone furoate** (DPI)	Without sufficient studies on children ≤5 years old
**Fluticasone propionate** (pMDI, standard particles, HFA)	50 (≥4 years old)
**Mometasone furoate** (pMDI, standard particles, HFA)	100 (≥5 years old)
**Ciclesonide** (pMDI, extra fine particles, HFA)	Without sufficient studies on children ≤5 years old

DPI: dry powder inhaler; HFA: hydrofluoroalkane propellant; ICS: inhaler corticosteroid; pMID: pressurized inhaler with fixed dose.

## Data Availability

Data sharing not applicable.
